# Load-dependent collagen fiber architecture data of representative bovine tendon and mitral valve anterior leaflet tissues as quantified by an integrated opto-mechanical system

**DOI:** 10.1016/j.dib.2019.105081

**Published:** 2020-01-03

**Authors:** Samuel V. Jett, Luke T. Hudson, Ryan Baumwart, Bradley N. Bohnstedt, Arshid Mir, Harold M. Burkhart, Gerhard A. Holzapfel, Yi Wu, Chung-Hao Lee

**Affiliations:** a Biomechanics and Biomaterials Design Laboratory (BBDL), School of Aerospace and Mechanical Engineering (AME), The University of Oklahoma, USA; b Center for Veterinary Health Sciences, Oklahoma State University, USA; c Department of Neurological Surgery, Indiana University School of Medicine, USA; d Division of Pediatric Cardiology, Department of Pediatrics, The University of Oklahoma Health Sciences Center, USA; e Division of Cardiothoracic Surgery, Department of Surgery, The University of Oklahoma Health Sciences Center, USA; f Institute of Biomechanics, Graz University of Technology, Austria; g Department of Structural Engineering, Norwegian University of Science and Technology, Norway; h Institute for Biomedical Engineering, Science and Technology (IBEST), The University of Oklahoma, USA

**Keywords:** Collagen fiber architecture, Microstructure, Polarized spatial frequency domain (pSFDI) imaging, Mechanical loading, Degree of optical anisotropy, Heart valve, Load-dependence

## Abstract

The data presented in this article provide load-dependent collagen fiber architecture (CFA) of one representative bovine tendon tissue sample and two representative porcine mitral valve anterior leaflet tissues, and they are stored in a MATLAB MAT-file format. Each dataset contains: (i) the number of pixel points, (ii) the array of pixel's *x*- and *y*-coordinates, (iii) the three acquired pixel intensity arrays, and (iv) the Delaunay triangulation for visualization purpose. This dataset is associated with a companion journal article, which can be consulted for further information about the methodology, results, and discussion of the opto-mechanical characterization of the tissue's CFA's (Jett *etal.* [1]).

**Table:** Specifications Table

Subject	Bioengineering
Specific subject area	Collagen Fiber Architecture of Collagenous Tissues
Type of data	MATLAB MAT-Files MATLAB Script File for Plotting the Data
How data were acquired	Instruments used for collagen fiber architecture quantification: (i) an in-house polarized spatial frequency domain imaging (pSFDI) device (ii) a commercial biaxial mechanical testing system (BioTester, CellScales) (iii) custom LabView program for imaging data acquisition (iv) custom Python script program for writing acquired image data into a MATLAB MAT-file format
Data format	(i) Raw (stored in the MATLAB MAT-files) (ii) Analyzed (data could be further analysed and visualized using the MATLAB script program pSFDI_process.m)
Parameters for data collection	Tissue type (bovine tendon and mitral valve anterior leaflets), testing temperature (37 °C), testing environment (under phosphate-buffered saline)
Description of data collection	Collagen fiber architecture (CFA) data for representative soft collagenous tissues (e.g., bovine tendon and porcine mitral valve anterior leaflets (MVALs)) were collected by using an integrated opto-mechanical instrument under emulated physiological conditions. Longitudinal strains (0%, 1%, 2%, 3%) were applied to the bovine tendon tissue, and various biaxial mechanical loads (unloaded and T_circ_:T_rad_=1:1, 1:0.25, 0.25:1) were considered for the porcine MVALs.
Data source location	School of Aerospace and Mechanical Engineering The University of Oklahoma Norman, Oklahoma, USA (35°12′36.6″N, −97°26′35.3″W)
Data accessibility	Repository name: Mendeley Data https://app.box.com/s/y882tawzfzosbp3qhismsjjmxjjirwcf
Related research article	Samuel V. Jett, Luke T. Hudson, Ryan Baumwart, Bradley N. Bohnstedt, Arshid Mir, Harold M. Burkhart, Gerhard A. Holzapfel, Yi Wu, and Chung-Hao Lee. “Integration of polarized spatial frequency domain imaging (pSFDI) with a biaxial mechanical testing system for quantification of load-dependent collagen architecture in soft collagenous tissues”, *Acta Biomaterialia*, (2020), **102**, 149-168, DOI: https://doi.org/10.1016/j.actbio.2019.11.028 [1]. (This Data-in-Brief submission is a co-submission of the research article.)

**Table:** 

**Value of the Data** • Describing the load-dependence of local, pixel-wise collagen fiber architectures (CFAs) in uniaxially-loaded tendon and biaxially-loaded mitral valve tissues • Providing novel information about the tissue microstructures by examining the differences between unloaded and mechanically-loaded tissues • Permitting researchers to build predictive models relating bulk mechanical loading to local microstructural changes in soft collagenous tissues • Facilitating ongoing/future investigations of the spatial heterogeneity of mitral valve leaflet tissues microstructural responses to loads, improving understanding of the tissue's physiological behaviors • Providing new research opportunities to the development tissue-engineered protheses, such as for heart valve surgical replacement, by mimicking the tissue mechanics and microstructure

## 1 Data

**Fig. 1:**
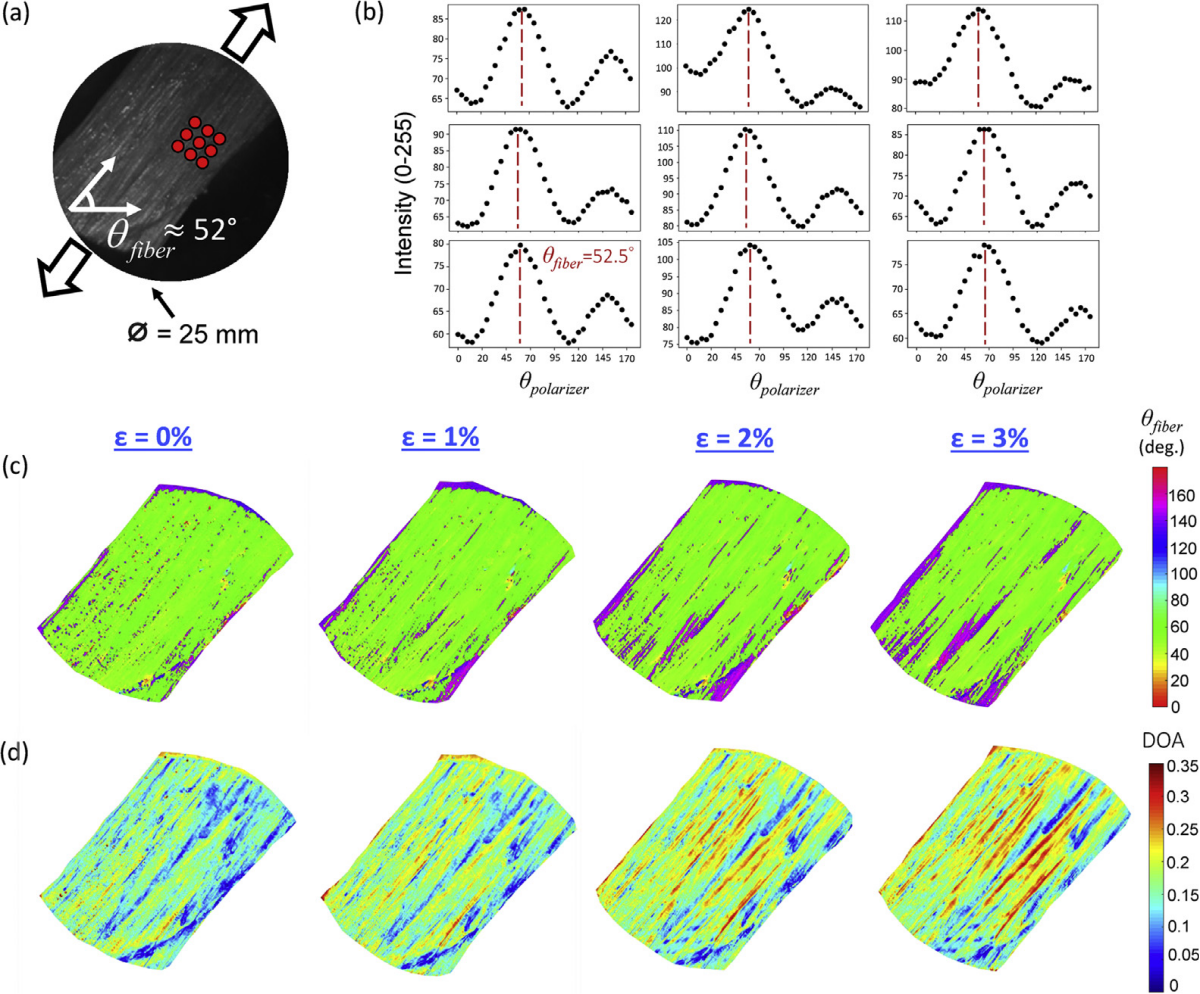
CFA quantifications for the bovine tendon specimen: (a) an experimental photo showing the longitudinal tendon axis of ∼52°, (b) DC intensity versus polarizer angle plots for the selected 3 × 3 grid pixel points, (see red dots in (a)). The angle corresponding to the peak intensity, indicated by the red dashed line, is the quantified fiber orientation angle *θ*_*fiber*_. Colormaps of (c) *θ*_*fiber*_ and (d) DOA at different longitudinal strain levels.

**Fig. 2:**
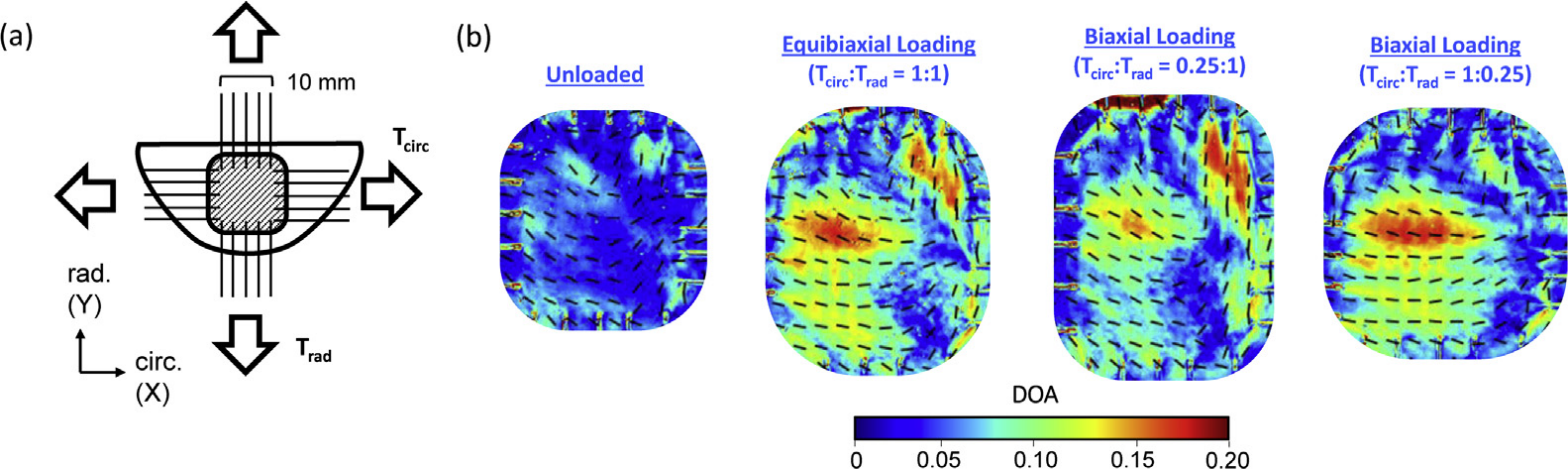
CFA quantifications for porcine mitral valve anterior leaflet specimen #1 (MVAL-1): (a) schematic of the biaxial mechanical testing in conjunction with polarized spatial frequency domain imaging-based collagen CFA quantification, and (b) the quantified collagen fiber orientation (black dashed lines) and the degree of optical anisotropy (colormaps) of the tissue at various loading conditions. Note that warmer colors denote a better aligned collagen fiber network.

**Fig. 3:**
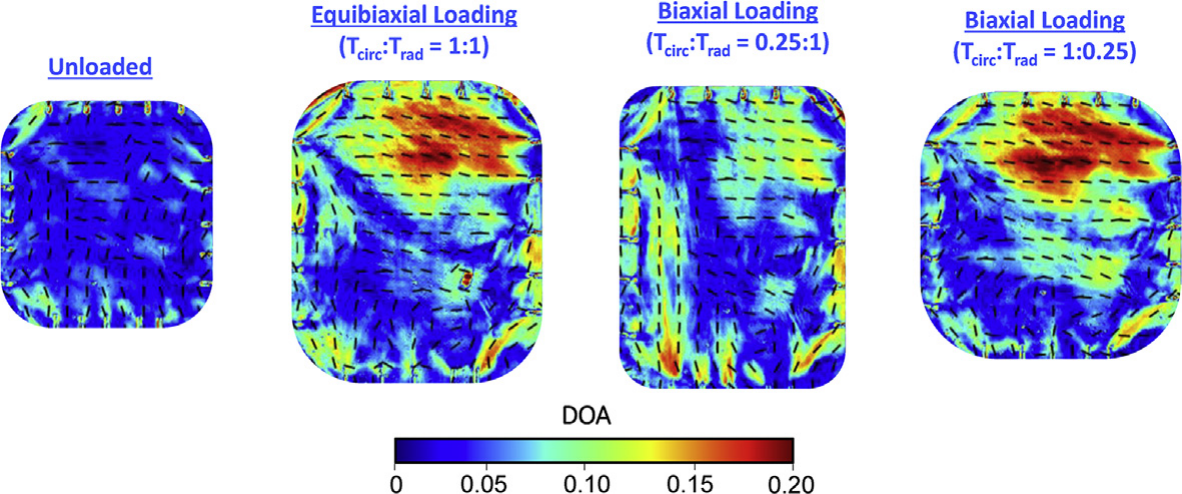
CFA quantifications for porcine mitral valve anterior leaflet specimen #2 (MVAL-2): the quantified collagen fiber orientation (black dashed lines) and the degree of optical anisotropy (colormaps) of the tissue at various loading conditions. Note that warmer colors denote a better aligned collagen fiber network.

The data presented in this document provide the load-dependent collagen fiber architecture (CFA) of one representative bovine tendon tissue specimen (thickness=1.25 mm, width=15 mm, length=40mm) and two representative porcine mitral valve anterior leaflet (MVAL) tissue specimens (effective testing region=10×10 mm, MVAL-1: thickness=0.75 mm, MVAL-2: thickness=0.87mm). The data sets in a MATLAB (MathWorks, MA) MAT-file format, as listed in Table 1, can be read by the provided MATLAB script program (pSFDI_process.m). Each data set contains: (i) the number of pixel points (nid_process, scalar), (ii) the array of pixel's x- and y-coordinates (coor, [2, nid_process]), (iii) the acquired pixel intensity arrays (II_1_grid, II_2_grid, II_3_grid, each array in [37 nid_process]), and (iv) the Delaunay triangulation for visualization purpose only (tri, [ntri, 3]). By using the same provided MATLB script program, each data set can be analyzed, and the CFA data, including the quantified collagen fiber orientation angle *θ*_*fiber*_ and the degree of optical anisotropy (DOA), can also be visualized (bovine tendon: Fig. 1, MVAL-1: Fig. 2, MVAL-2: Fig. 3).

**Table 1:** Filenames of the load-dependent CFA data sets regarding the investigated tissue specimens.

Tissue Specimen	Testing Condition	MATLAB MAT-Filename
Bovine Tendon	Unloaded, 0% longitudinal strain	Tendon_0_percent_raw.mat
	Loaded, 1% longitudinal strain	Tendon_1_percent_raw.mat
	Loaded, 2% longitudinal strain	Tendon_2_percent_raw.mat
	Loaded, 3% longitudinal strain	Tendon_3_percent_raw.mat
MVAL Specimen 1 (MVAL-1)	Unloaded	MVAL-1_0_to_0_raw.mat
	Equibiaxial tension, *T*_*circ*_:*T*_*rad*_=1:1	MVAL-1_1_to_1_raw.mat
	Non-equibiaxial tension, *T*_*circ*_:*T*_*rad*_=1:0.25	MVAL-1_1_to_025_raw.mat
	Non-equibiaxial tension, *T*_*circ*_:*T*_*rad*_=0.25:1	MVAL-1_025_to_1_raw.mat
MVAL Specimen 2 (MVAL-2)	Unloaded	MVAL-2_0_to_0_raw.mat
	Equibiaxial tension, *T*_*circ*_:*T*_*rad*_=1:1	MVAL-2_1_to_1_raw.mat
	Non-equibiaxial tension, *T*_*circ*_:*T*_*rad*_=1:0.25	MVAL-2_1_to_025_raw.mat
	Non-equibiaxial tension, *T*_*circ*_:*T*_*rad*_=0.25:1	MVAL-2_025_to_1_raw.mat

## 2 Experimental design, materials, and methods

### 2.1 Tissue retrieval and storage

Bovine tendon and porcine hearts were acquired from a local USDA-approved slaughterhouse (Country Home Meats Co., Edmond, OK) and frozen in a standard freezer at −20 °C for storage purpose. Previous studies have shown effectiveness of this tissue storage protocol for maintaining the tissue integrity, microstructures and mechanics [[2], [3], [4], [5], [6], [7]].

### 2.2 Tissue dissection and preparation

For tendon tissue preparation, the central region of bovine tendon was excised into thin tissue sample (width=15 mm, length=40 mm, thickness=1.25 mm), with care taken to exclude the synovial sheath membrane enclosing the tendon and align the strip length direction with the native tendon axis (Fig. 1a). For leaflet acquisition, porcine hearts were slowly thawed in a saline bath at room temperature and were then dissected to obtain the mitral valve anterior leaflet (MVAL) tissue specimens with an effective testing size of 10×10 mm (Fig. 2a). The dissected tissue samples were placed in a labelled container of phosphate-buffered saline (PBS), and stored in a refrigerator at 4 °C until testing (within two days).

### 2.3 Opto-mechanical testing – polarized spatial frequency domain imaging of the tissue samples

**Fig. 4:**
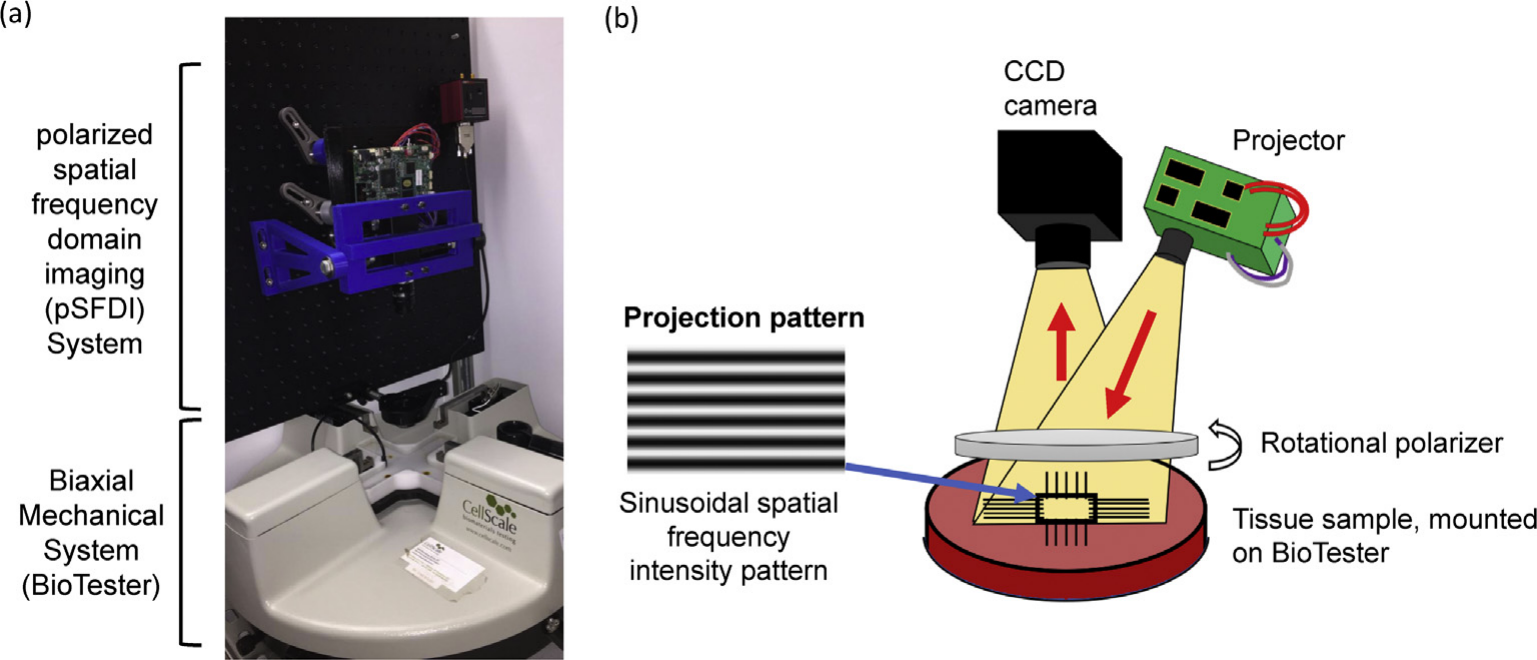
(a) Trimetric view of the integrated opto-mechanical system used in the collection of the presented data, and (b) schematic of a co-polarized pSFDI system, showing the optical components (DLP project, CCD camera, rotational polarizer), the passage of light, and the scattering from the fibrous tissue microstructure.

For the quantification of the load-dependent collagen fiber architecture (CFA) of both the bovine tendon and MVAL tissue specimens, an integrated instrument (Fig. 4a), which combines a commercial biaxial mechanical testing system (BioTester, CellScale, Canada) and an in-house polarized spatial frequency domain imaging (pSFDI) device, was used.

In brief, the bovine tendon sample was then mounted to the BioTester via the CellScale clamp mounting fixture and subjected to various longitudinal strains (0%, 1%, 2% and 3%) along the tendon tissue's length direction (Fig. 1a). At each strain state, the collagen fiber orientation and the DOA within the sample were quantified using the integrated instrument with a spatial frequency of *f*_*x*_=0.20 mm^−1^. Sample hydration was maintained by soaking the sample in PBS solution during the imaging tests.

For mitral valve leaflet testing, the entire anterior leaflet tissue samples were excised from the porcine mitral heart valve were mounted to the BioTester using the CellScale BioRakes fixture to create an effective testing region of 10×10 mm (Fig. 2a). The MVAL tissue sample's circumferential and radial directions were aligned with the *x-* and *y-*axes of the tester, respectively, during mounting. The tissues were then immersed in a PBS solution at 37 °C for the duration of mechanical testing to emulate the valve's physiological conditions. Prior to applying the mechanical testing protocols, the tissue samples were preconditioned to restore their *in vivo* functional state using a standard force-controlled preconditioning protocol with a targeted maximum force of 1 N applied in both the circumferential and radial directions associated with the tissue's collagen fiber networks [8,9]. The targeted loading of 1 N was determined based on an assumed physiological membrane tension of 100 N/m [10,11] and a 10 mm effective edge length. The MVAL tissue sample was subjected to various biaxial loads: *T*_*circ*_*:T*_*rad*_=1:1 (equibiaxial loading), *T*_*circ*_*:T*_*rad*_=1:0.25, and *T*_*circ*_*:T*_*rad*_=0.25:1, where *T*_*circ*_ and *T*_*rad*_ are the membrane tensions applied in the MVAL tissue's circumferential and radial directions, respectively. During pSFDI imaging tests, a spatial frequency of *f*_*x*_=0.27 mm^−1^ was adopted.

### 2.4 pSFDI imaging procedure

The pSFDI imaging technique combines the ability of co-polarized imaging to quantify the birefringent fiber structures with the depth-discrimination capabilities of SFDI. Interested readers can refer to more details in Refs. [1,[12], [13], [14]]. The pSFDI system (Fig. 4b) utilized an LED-driven, micromirror-based pattern projection system (Texas Instruments, Dallas, TX) with a projection wavelength of 490 nm (cyan) and a 5-Megapixel CCD camera (Basler, Germany) with lens of *f/1.9* and an exposure time of 50 ms. For controlled rotational polarization, our pSFDI system employed a nanoparticle linear polarizer with a diameter of 25 mm mounted into a rotational servo motor with a 0.1° resolution (Thorlabs, Newton, NJ). During pSFDI imaging, three phase-shifted images were projected sequentially, through a polarizer at angle *θ*_*polarizer*_, and onto the tissue sample. The reflected light from the sample passed back through the same polarizer and was captured by the CCD camera. This projection-capture sequence was repeated at each of the 37 discrete polarization increments (5° increments from 0° to 180°) using an in-house LabView controlling program (National Instruments, Austin, TX).

### 2.5 pSFDI image data analysis – quantification of fiber orientation angle *θ*_*fiber*_ and degree of optical anisotropy (DOA)

After pSFDI imaging, the 37 phase-shifted images were first smoothed via convolution with a normalized 5 × 5 uniform kernel and were then combined at each pixel and polarization state to obtain the resultant DC and AC intensities: the DC intensity *I*_*DC*_ which provides equal weighting for each reflected photon by representing the conventional diffuse reflectance image, and the AC intensity *I*_*AC*_, which signifies the differences between the spatially-modulated intensity patterns.

(1) $$I_{\mathrm{DC}}=\frac{I_{0{}^{\circ}}+I_{120{}^{\circ}}+I_{240{}^{\circ}}}{3},\;and\;I_{\mathrm{AC}}=\frac{\sqrt{2}}{3}\sqrt{{\left(I_{0{}^{\circ}}-I_{120{}^{\circ}}\right)}^{2}+{\left(I_{120{}^{\circ}}-I_{240{}^{\circ}}\right)}^{2}+{\left(I_{240{}^{\circ}}-I_{0{}^{\circ}}\right)}^{2}}.$$

Herein, *I*_0°_, *I*_120°_, and *I*_240°_ are the pixel-wise intensity corresponding to the three phase shifts, respectively. The global maxima of these intensity functions occurs when the polarizer transmission axis *θ*_*polarizer*_ is parallel to and perpendicular to the fiber orientation angle *θ*_*fiber*_, respectively (Fig. 1b).

Quantitatively, the birefringent reflected intensity *I*_*out*_ of a group of collagen fibers (Fig. 4b) can be described by the following 3-term Fourier cosine series:

(2) $$\frac{I_{\mathrm{out}}}{\tau_{\mathrm{sys}}}=a_{0}+a_{2}\left[2\left(\theta_{\mathrm{fiber}}-\theta_{\mathrm{polarizer}}\right)\right]+a_{4}\left[4\left(\theta_{\mathrm{fiber}}-\theta_{\mathrm{polarizer}}\right)\right],$$

where *τ*_*sys*_ is a bulk systemic coefficient encompassing non-birefringent intensity modifiers, such as the aperture of the camera, and *a*_0_, *a*_2_, and *a*_4_ are the three Fourier coefficients. The magnitudes of the optical anisotropies provide a means of quantitatively examining the local dispersion of the collagen fibers, which is expressed in the degree of optical anisotropy (DOA), i.e.,

(3) $$DOA=\frac{a_{2}+a_{4}}{a_{0}+a_{2}+a_{4}}.$$

Please refer to more details about the step-by-step algorithmic procedures in Section 2.3 of [1].
